# Advancing Ischemic Stroke Prognosis: Key Role of MiR-155 Non-Coding RNA

**DOI:** 10.3390/ijms26093947

**Published:** 2025-04-22

**Authors:** Catherine Hering, Gloria M. Conover

**Affiliations:** Department of Medical Education, College of Medicine, Texas A&M University, Bryan, TX 77807, USA; catherinehering@tamu.edu

**Keywords:** apoptosis, ischemic stroke, microglia polarization, microRNAs, miR-155, neuroinflammation, neuroprotection, non-coding RNAs, oxidative stress, SIRT1, stroke therapeutics

## Abstract

Ischemic stroke (IS) is the leading cause of long-term disability and the second leading cause of death worldwide. It remains a significant clinical problem because only supportive therapies exist, such as thrombolytic agents and surgical thrombectomy, which do not restore function. Understanding the molecular pathogenesis of IS, including dysfunction in oxidative homeostasis, apoptosis, neuroinflammation and neuroprotection, is crucial to developing therapies. Non-coding RNAs (ncRNAs) are master regulators, and one ncRNA that stands out is miR-155, a pro-inflammatory micro-RNA elevated in stroke. This review addresses the biological mechanisms reported in the literature that support using miR-155 as a biomarker and therapeutic agent to treat IS in patients.

## 1. Introduction

Stroke is a serious cardiovascular cause of morbidity and mortality [1,2,3,4,5,6]. In 2022, stroke was the third leading cause of disability worldwide and the second leading cause of death, with 7.5 million people diagnosed with acute ischemic stroke (IS) [6,7,8]. In the US, that same year, 1 in 6 people with cardiovascular disease died of stroke [8]. Globally, the incidence of stroke is 15 million people per year, and of these, 5 million people die from stroke and another 5 million will become disabled [4,5,6,9,10]. Risk factors that contribute to poor stroke prognosis span from lifestyle and environmental toxins to various accompanying comorbidities. Major modifiable risk factors for stroke are hypertension, diabetes mellitus, smoking, hyperlipidemia, obesity, poor diet/nutrition, and lack of exercise [11,12,13]. Of the modifiable risk factors, the highest is hypertension (54%) [14,15] while non-modifiable stroke risk factors are age, sex, and ethnicity [16].

Over 70% of strokes occur in patients over 65 years old, while ~75% of strokes occur in patients over 64 with males overall exhibiting a higher incidence (~33%) and prevalence (~41%) as compared to females [17,18]. Remarkably, non-Hispanic African Americans have ~2.2 times higher incidence of stroke as compared to Caucasian patients [19]. However, women over 75 years old have more strokes compared to men of the same age. After menopause, women have elevated FSH which promotes lipogenesis and fat storage, increasing androgen levels [20]. High-risk pregnancy, contraceptive use and substance abuse increase the risk for stroke in younger patients [21].

Non-coding RNAs (ncRNAs) are RNAs that do not encode functional proteins and were originally thought to regulate post-transcriptional gene expression [22]. Interestingly, many reports have shown that ncRNAs expression are detected when disease symptoms occur, making ncRNAs attractive disease biomarkers [23]. For example, deletions of miR-15a and miR-16 were found in 68% of chronic lymphocytic leukemia patients, and 296 differentially expressed miRNAs were identified in a rat middle cerebral artery occlusion (MCAO) stroke model. These studies revealed a promising clinical use of micro-RNAs (miRNAs) as plausible markers monitoring different stages of stroke within miRNA-containing exosomes or extracellular vesicles (EVs) [24,25,26].

miRNAs are conserved non-coding single-stranded RNAs that temporally regulate tissue-specific gene expression [27,28]. As powerful regulators of cellular activity, miRNAs drive cell differentiation and developmental programs [29,30]. Today, several ncRNA therapeutics are in clinical trials for cancer (liver, lymphoma and colorectal), endometriosis, and Alzheimer’s disease [27,30]. Active processed miRNAs range between 19 and 25 base pairs in length while long non-coding RNAs (lncRNAs) can reach ~200 base pairs [31]. Although both ncRNAs are prone to degradation by RNAases, circular RNAs (circRNAs) are less so due to their stability [32]. In this review, we and others propose that stroke-associated ncRNAs modulate stroke disease stage and progression. Our discussion is focused on miR-155, a proinflammatory miRNA in strokes, with pivotal roles in oxidative stress, apoptosis, neuroinflammation, and microglia polarization. Here, we compiled compelling experimental evidence that profiles miR-155 as a promising prognosis biomarker and therapeutic inhibitor.

## 2. Overview of Stroke Pathophysiology

**Figure 1:**
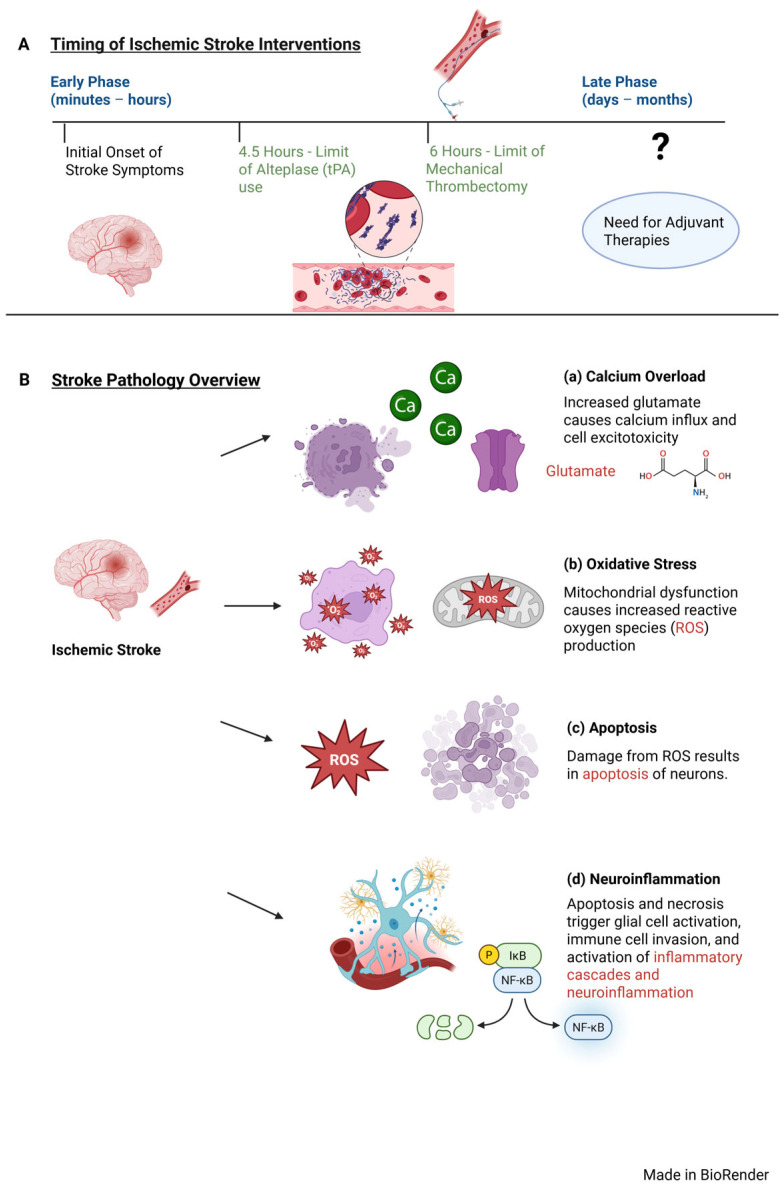
**Timeline of Therapeutic Intervention in Treating Ischemic Stroke.** (**A**) At the onset of stroke symptoms, there is a 4.5-h window to use alteplase (tPA), a powerful thrombolytic that works by converting plasminogen to plasmin to dissolve fibrin clots, and a 6-h window to use mechanical thrombectomy, a procedure where a catheter is physically inserted into a blood vessel to remove clots. Therapeutic options beyond these time windows are limited. (**B**) The overview of stroke pathology shows the ischemia triggers activation of simultaneous molecular cascades, (**a**) excitotoxicity and calcium overload, (**b**) oxidative stress, (**c**) apoptosis, and (**d**) neuroinflammation.

Stroke is typically defined as an acute neurological deficit caused by vascular damage to the CNS [33,34,35]. The majority of IS cases reported in the United States (87%) occur when a clot forms in a blood vessel in the brain [36]. In contrast, hemorrhagic stroke occurs (13%) when blood pools in the cerebral parenchyma that compresses adjacent brain structures [37,38]. Routine tools in the clinical settings are unreliable at predicting stroke outcomes [39]. It is well-documented that insufficient cerebral blood flow in IS triggers neurological dysfunction by disrupting the brain–blood barrier and altering oxidative stress, apoptosis, and neuroinflammation [40]. The initial ischemia in a stroke triggers multiple signaling cascades that perturb homeostasis leading to neuron and vascular dysfunction (Figure 1) [41,42,43,44]. Apoptosis from ischemia causes membrane leakage of cell contents and pump malfunction, causing build-up of excitatory glutamate, activation of NMDA and AMPA glutamate receptors, and influx of calcium (Figure 1B) [45,46,47]. The influx of calcium causes mitochondria membrane swelling, exacerbating oxidative stress and elevating production of reactive oxygen species (ROS) (Figure 1B) [48,49,50,51,52]. This damage leads to necrosis, increased neuroinflammation caused by activated glial cells and invasion of leukocytes, and monocytes in the brain (Figure 1B) [53,54,55,56,57]. Activated microglia secrete pro-inflammatory cytokines, while hypoxia increases NF-κB levels and associated hypoxia-inducible proteins (Figure 1B) [58,59,60,61].

## 3. Stroke Management Timing Considerations

Despite the high disease burden of stroke worldwide, therapeutic options are limited [62]. The first line of treatment for IS in adults is alteplase (tPA), a thrombolytic agent that breaks down a thromboembolic clot [63]. However, tPA has a very narrow time window to be safely prescribed. tPA cannot be administered safely to stroke patients ~4.5 h after initial symptom onset, or from the time of the last known normal state of the patient [62,63]. In infants, hypoxic–ischemic brain damage (HIBD), a brain injury caused by lack of adequate oxygen and blood flow, is a major cause of long-term neurological deficits, often resulting in cerebral palsy, mental retardation and epilepsy. Use of alteplase (tPA) beyond this 4.5-h window increases the risk of a re-bleed and can cause a new hemorrhagic stroke [64]. Moreover, tPA therapy is contraindicated for patients with high blood pressure (>185 mmHg systolic blood pressure or >110 mmHg diastolic blood pressure), a diagnosis of prior stroke in the last 3 months, or recent use of anticoagulants (e.g., Warfarin or factor Xa inhibitor Eliquis) [65]. Surgical mechanical thrombectomy is the other standard treatment for IS caused by an occluding thromboembolism. However, like with tPA, mechanical thrombectomy also has a narrow window of therapeutic use and can only be used within 6 h of symptom onset [66]. With such a narrow window of opportunity for standard of care, there is an urgent clinical need to develop adjuvant therapies to treat IS patients (Figure 1A).

## 4. Non-Coding RNAs: Promising Biomarkers for Stroke Progression

As mentioned above, non-coding RNAs, are a class of conserved RNAs that are not translated into proteins, which surprisingly constitute up to 97% of the entire human genome [67]. These RNAs regulate protein expression by degrading or inhibiting mRNA translation or by directly silencing transcription. For example, ncRNAs can (1) target the 3′ or 5′ untranslated region of specific mRNAs to cause mRNA degradation, repression, or translation, (2) act as mediators of intracellular communication by serving as hormones or being secreted in EVs, (3) directly targeting toll-like receptors (TLRs) to activate signaling pathways and immune responses, (4) cooperatively/competitively interact to enhance or silence targets, and (5) act as pri-miRNAs, encoding peptides that can regulate mature miRNA expression [27,68,69,70,71,72,73,74,75,76,77,78].

**Figure 2:**
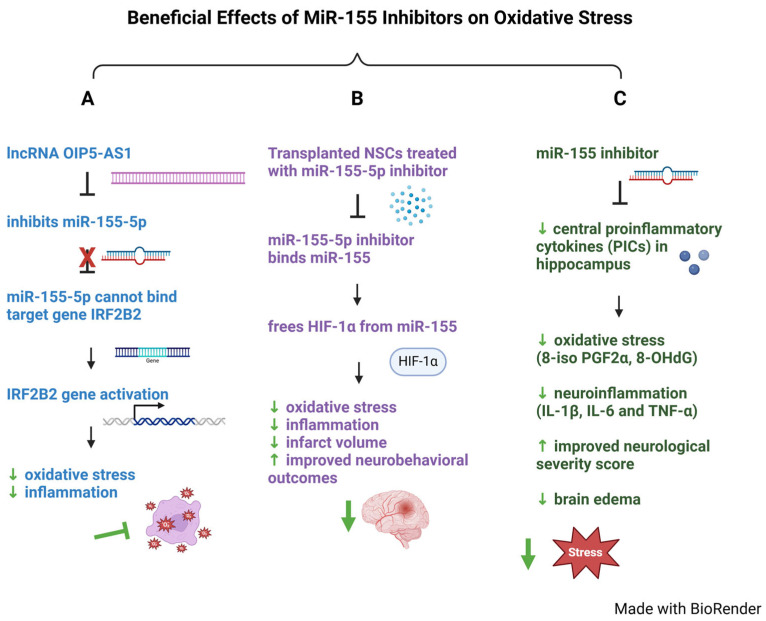
**Beneficial Effects of MiR-155 Inhibitors on Oxidative Stress.** miR-155 inhibitors reduce oxidative stress. (**A**) LncRNA OIP5-AS1 inhibits miR-155-5p, preventing miR-155-5p from binding its target gene, IRF2B2, permitting IRF2B2 to reduce oxidative stress and inflammation. Note that “miR-155” refers to the whole miRNA molecule, “miR-155-5p” specifically denotes the mature form of miR-155 derived from the 5′ arm precursor molecule. (**B**) Neural stem cells (NSCs) treated with miR-155-5p inhibitor frees HIF-1α from miR-155, resulting in decreased oxidative stress, inflammation, infarct volume, and improved neurobehavioral outcomes. (**C**) miR-155 inhibitors decreased PICs in the hippocampus, resulting in decreased oxidative stress (8-iso PGF2α, 8-OHdG), neuroinflammation (IL-1β, IL-6 and TNF-α), improved neurological severity score and brain edema. miR-155 inhibitors have potential to decrease oxidative stress, apoptosis, neuroinflammation, and increase neuroprotection in ischemic stroke patients. It is predicted that miR-155 inhibitors will have some effect in restoring blood flow in the infarct area of the stroke, delaying neuronal death. Green arrows indicate beneficial outcomes in ischemic stroke.

**Figure 3:**
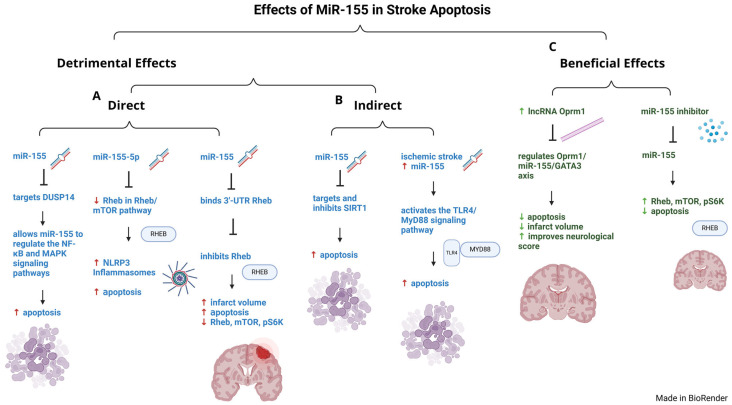
**Effect *of MiR-155 in* Stroke Apoptosis.** miR-155 promotes apoptosis in ischemic stroke. (**A**) Detrimental effects (direct)—miR-155 targets DUSP14, thereby regulating the NF-κB and MAPKs signaling pathways to worsen apoptosis. miR-155-5p decreases Rheb in the Rheb/mTOR pathway, resulting in increased NLRP3 inflammasomes and apoptosis. miR-155 binds the 3′-UTR of Rheb, inhibiting Rheb to promote increased apoptosis, infarct volume, and decreasing Rheb. (**B**) Detrimental effects (indirect)—miR-155 targets and inhibits SIRT1 to promote apoptosis. Ischemic stroke increases miR-155 which activates the TLR4/MyD88 signaling pathways, increasing apoptosis. (**C**) Beneficial Effects—Increased levels of lncRNA Oprm1 regulates the Oprm1/miR-155/GATA3 axis, reducing apoptosis and injury. miR-155 inhibitors reduce apoptosis, infarct volume, and increase Rheb, mTOR, and pS6K expression. Green arrows indicate beneficial outcomes, and red arrows indicate harmful outcomes in ischemic stroke.

**Figure 4:**
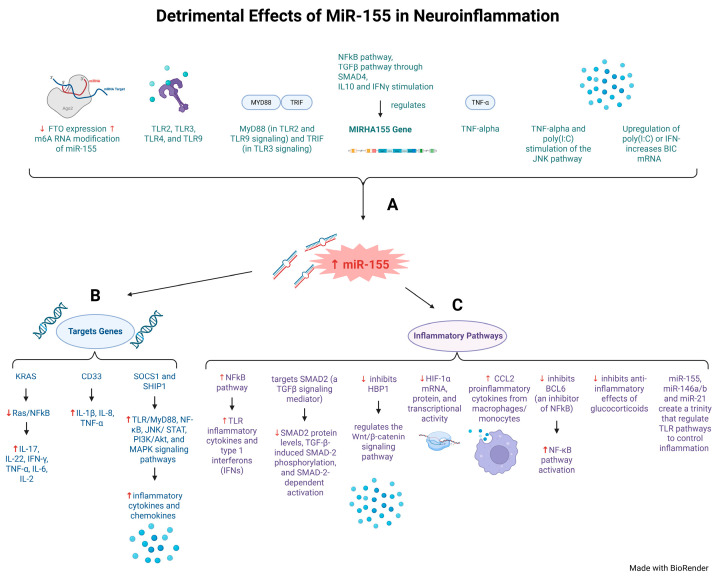
**Detrimental effects of miR-155 in neuroinflammation.** miR-155 plays several roles in neuroinflammation. (**A**) miR-155 can be regulated through RNA modification, TLR signaling, and through inflammatory pathways through the MIRHA155 gene that controls miR-155 expression (green text). (**B**) miR-155 can activate or inhibit specific target genes, including KRAS, CD33, SOCS1 and SHIP1, to further promote inflammation (blue text). (**C**) miR-155 directly targets inflammatory pathways, including NF-kB, to promote inflammation (purple text). Red arrows indicate harmful outcomes in ischemic stroke.

**Figure 5:**
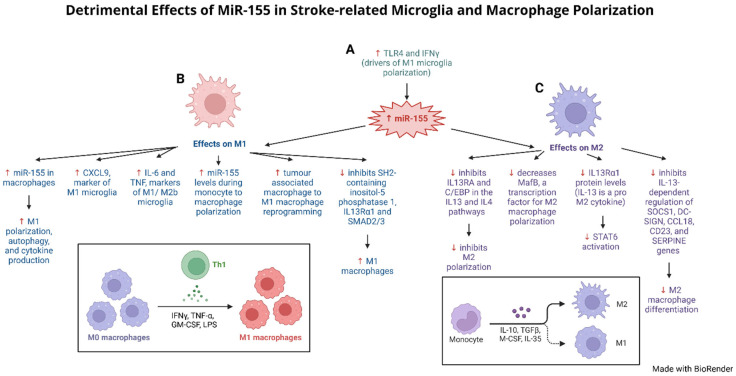
**Detrimental Effects of MiR-155 in Stroke-related Microglia and Macrophage Polarization.** (**A**) TLR4 and IFNγ, drivers of M1 microglia polarization, both increase miR-155 levels (green text). (**B**) miR-155 acts to promote M1 pro-inflammatory microglia polarization through multiple pathways. Increased miR-155 enhances cytokine production, autophagy, and M1 polarization, detected by CXCL9 (M1 marker) and IL-6 and TNF (M1/M2b markers). miR-155 levels are elevated during monocyte to macrophage differentiation and are increased when tumor-associated macrophages are reprogrammed to M1 macrophages. This is potentially through miR-155 targeting and suppressing SH2-containing inositol-5-phosphatase 1, IL13Rα1 and SMAD2/3, which promotes M1 macrophages (blue text). (**C**) miR-155 inhibits M2 anti-inflammatory microglia polarization through multiple pathways. miR-155 inhibits M2 polarization by inhibiting IL13RA and C/EBP in the IL13 and IL4 pathways, and by suppressing MafB, a transcription factor for M2 macrophage polarization. miR-155 also directly targets IL13Rα1 (a pro-M2 cytokine), causing reduced STAT6 activation, and affects IL-13-dependent regulation of SOCS1, DC-SIGN, CCL18, CD23, and SERPINE genes involved in differentiating M2 macrophages (purple text). Red arrows indicate harmful effects/outcomes in ischemic stroke.

Ongoing research shows that ncRNA dysregulation underlies the biology of many chronic diseases [79,80,81,82,83,84,85,86,87,88,89,90,91,92,93]. Notably, single ncRNAs act to restore or degrade neural plasticity, axonal regeneration, vascular reorganization and stem cell activation [92]. Hence, it is important to understand the relationship of molecular targets of specific types of ncRNAs. Remarkably, miR-155 is an excellent candidate given its central role in neuroinflammation and neurodegeneration [68,92]. miR-155, originally discovered in 1997 as a conserved ncRNA in chicken, mice and humans [94], plays a key role in B-cell/macrophage inflammation [94]. miR-155 was reported to also be involved in vascular remodeling as it was found to be abundant in the cerebrovascular endothelium, astrocytes and microglia [95]. Early uncontrolled activation of neuroinflammation during stroke leads to rapid brain dysfunction [96,97,98,99]. We and others propose, given its profile, that miR-155 is a potent regulator that could modulate cellular damage and limit the rapid progression of disability during stroke. Here we present experimental evidence on how miR-155 constitutes an attractive, reliable time-sensitive biomarker, and that inhibition could potentially mitigate oxidative stress, apoptosis, and neuroinflammation, while simultaneously increasing neuroprotection (Figure 2, Figure 3, Figure 4 and Figure 5, Table 1).

### 4.1. Role of MiR-155 in Oxidative Stress

The therapeutic potential for miR-155 to modulate stroke-induced oxidative stress (Figure 2, Table 2) was established when it was realized that miR-155-5p was highly expressed during ischemia/reperfusion injuries, and that miR-155-5p suppression improved stroke outcomes [116]. Consistent with these findings, lncRNA OIP5-AS1 was recognized as a beneficial molecule, since when it was upregulated it suppressed oxidative stress. In comparison, the interferon regulatory factor 2 binding protein 2 (IRF2BP2) is a transcriptional corepressor which acts as a protective factor in oxidative stress and inflammation in ischemic stroke [127,128]. Based on these findings, a bioinformatic prediction study identified binding sites between lncRNA OIP5-AS1, miR-155-5p and IRF2BP2. This suggests a functional relationship between lncRNA OIP5-AS1, miR-155-5p, IRF2BP2 (Figure 2A, Table 2) [129].

Studies using the lncRNA OIP5-AS1 in a cell model of IS using HMC3 cells (microglia) and SH-SY5Y cells (human neuroblastoma) exposed to oxygen-glucose deprivation/reoxygenation (OGD/R) mimic stroke conditions by stimulating restricted blood flow while depriving cells of oxygen and glucose, and subsequently restoring them to simulate return of blood flow. This is achieved by placing cells in a sugar-free medium in a gas chamber with low oxygen for 2 h, followed by a return to a normal sugar medium and oxygen levels for 12 h [129,136]. In this situation, the elevated reactive oxygen species (ROS), malondialdehyde (MDA) markers, and reduced superoxide dismutase (SOD) (antioxidant) showed that stroke-injured brains had decreased lncRNA OIP5-AS1 levels and increased oxidative stress (Figure 2A, Table 2). Stroke injury increases miR-155-5p levels with associated increases in oxidative stress, but overexpression of lncRNA OIP5-AS1 directly binds and inhibits miR-155-5p, reducing oxidative stress and injury [129,137]. These findings confirm previous studies where lncRNA OIP5AS1 upregulation suppressed oxidative stress in ischemic/reperfusion injuries in cardiomyocytes [138]. IRF2BP2, which is thought to be a protective protein against oxidative stress, is a downstream target of miR-155-5p. When miR-155-5p can bind to its target IRF2BP2, it results in increased oxidative stress. However, when upregulation of IRF2BP2, in the OGD/R-induced HMC3 and SH-SY5Y cell model, overcomes miR-155-5p’s inhibition, this leads to reduction in oxidative stress (Figure 2A, Table 2) [129].

Examining the effects of inhibiting miR-155-5p expression in neural stem cell (NSC) transplants the connection between miR-155 and oxidative stress during stroke (Figure 2B, Table 2) [132]. To investigate how HIF-1α is regulated by miR-155-5p, researchers focused on hypoxia-inducible factor 1α (HIF-1α), a transcription factor and master regulator of genes involved in maintaining oxygen homeostasis [139], by using the middle cerebral artery occlusion model (MCAO). This well-characterized model for stroke injury [140] simulates reperfusion similar to those found with tissue plasminogen activator (tPA) therapy, creating a setting for testing new stroke therapies [140].

When the miR-155-5p expression was inhibited in neural stem cells (NSCs) by using an miR-155-5p inhibitor (inhibitor nucleotide sequence 5′-ACCCCTATCACAATTAGCATTAA-3′ that was cloned into GV280 lentiviral vector) [132] the data showed that miR-155-5p directly targets HIF-1α and negatively regulates its expression by promoting oxidative stress. In contrast, when investigators used the miR-155-5p inhibitor in NSCs, this reversed miR-155-5p’s inhibition of HIF-1α, resulting in significantly reduced oxidative stress and inflammation, decreasing infarct volume and improving rat neurobehavioral outcomes (Figure 2B, Table 2). Taken together, these data indicate that a miR-155 inhibitor might be a promising neuroprotective agent against stroke.

The central pro-inflammatory cytokine (PIC) is a known marker used to track neurological deficits after transient global ischemia induced by cardiac arrest (Figure 2C, Table 2) [133]. In a MCAO study, transient global ischemia in the brain was created by inducing cardiac arrest in mice followed by cardiopulmonary resuscitation. To investigate whether miR-155 inhibition could improve oxidative stress and neuroinflammation in the hippocampus, the levels of oxidative stress markers 8-isoprostaglandin F2α (8-iso PGF2α, an indicator of oxidative stress), and 8-hydroxy-2′-deoxyguanosine (8-OHdG, indicator of protein oxidation), and neuroinflammatory markers IL-1β, IL-6 and TNF-α were measured (Figure 2C, Table 2). Results showed that the miR-155 inhibitor significantly reduced upregulation of hippocampus pro-inflammatory cytokines (PICs), decreased oxidative stress, improved neurological severity score and reduced brain edema, making miR-155 inhibitors a possible therapeutic for IS (Figure 2C, Table 2).

### 4.2. Role of MiR-155 in Stroke-Related Apoptosis

Neuronal apoptosis is another feature for cerebral ischemic injury that spans ischemia-induced ROS elevation and mitochondria dysfunction through a canonical mitochondria permeability transition pore (MPTP) [141]. Importantly, it has been recognized that the inhibition of miR-155 may be leveraged to treat IS (Figure 3C, Table 2). In vitro oxygen-glucose deprivation/reperfusion (OGD/R) cell models and in vivo hypoxic–ischemic brain damage (HIBD) animal models have showed that miR-155 inhibits SIRT1 (sirtuin1), a nicotinamide adenosine dinucleotide (NAD)-dependent protein deacetylase, resulting in increased apoptosis and inflammation (Figure 3B, Table 1) [115]. In this cell model of OGD/R-treated PC12 cells and microglia (BV2) cells, miR-155 inhibitors decreased apoptosis. However, when SIRT1 was silenced, miR-155 inhibitors were dysfunctional, indicating that SIRT1 was required to reduce apoptosis. These findings were confirmed in the in vivo rat HIBD model. Together, this indicates that miR-155 effectively targets and inhibits SIRT1 to promote apoptosis in IS (Figure 3B, Table 1).

The therapeutic potential of miR-155-5p inhibition is supported by additional evidence (Figure 3A). Results in a study showed that miR-155-5p was upregulated in a rat middle cerebral artery occlusion/reperfusion (MCAO/R) and a OGD/R SH-SY5Y cell model [116]. In both models, miR-155-5p worsened ischemic neuronal apoptosis while inhibition of miR-155-5p improved it. miR-155-5p was shown to target and inhibit DUSP14 by binding to its 3′UTR, thereby preventing DUSP14 from inhibiting the NF-κB and MAPKs signaling pathways, resulting in increased apoptosis (Figure 3A, Table 1). Neuronal apoptosis of VECs plays a vital role in the pathophysiology of stroke through the toll-like receptor on microglia (TLR4) and its adaptor protein (MyD88) [117]. miR-155 regulates the apoptosis of VECs having an impact on cerebral IS apoptosis [142,143]. A relation between miR-155, apoptosis of VECs, and the TLR4/MyD88 signaling pathway was found when increased miR-155 levels activated the TLR4/MyD88, worsening the inflammation (Figure 3B, Table 1).

A cadre of miRNAs cooperate to synchronously integrate various regulatory pathways in the brain [144] as described. The apoptosis-associated miRNAs in a rat cerebellar ischemic alcoholism model found that miR-126 and miR-155 were both increased with apoptosis [144]. In an oxygen-glucose deprivation cell model using N2a cells, researchers investigated the mechanism of action for lncRNA Oprm1 following IS [134]. Researchers found that overexpression of lncRNA Oprm1 ameliorated apoptosis, through a lncRNA Oprm1/miR-155/GATA3 axis, by significantly decreasing infarct size and improving neurological score (Figure 3C, Table 2).

In line with these studies, EVs derived from choroid plexus epithelial (CPE) cells exhibited high levels of miR-155-5p [118]. This indicates that the neuron CPE derived EVs were likely able to deliver miR-155-5p to stroke lesions in the brain. In an OGD/R cell model, miR-155-5p overexpression from the EVs resulted in increased apoptosis, autophagic protein expression, reduced neuron viability, and activation of NLRP3 inflammasomes to worsen cell injury (Figure 3A, Table 1). miR-155-5p promoted apoptosis by suppressing Rheb expression, an important component of the mTOR pathway that promotes cell growth, and by promoting NLRP3-mediated inflammasomes to worsen ischemic damage.

The relationship between miR-155, apoptosis and the Rheb/mTOR was recognized in a MCAO rats and OGD/R cell culture models, when it was reported that miR-155 binds to the 3′-UTR of Rheb to inhibit its expression (Figure 3A, Table 1) [135]. High cerebral infarct volumes and apoptosis were associated with increased miR-155 and decreased Rheb, mTOR, and pS6K, while treatment with miR-155 inhibitors were protected with reduced apoptosis and increased Rheb, mTOR, and p-S6K expression (Figure 3A, C, Table 1). These data indicate that miR-155 inhibitors prevent neuronal apoptosis through the Rheb/mTOR pathway.

### 4.3. Role of MiR-155 in Neuroinflammation

After stroke onset, the pro-inflammatory master regulator nuclear factor-kappa B (NFkB) triggers heterogenous brain tissue damage that can lead to substantial disability. miR-155 induces neuroinflammation by blocking anti-inflammatory processes through multiple targets (Figure 4B, C, Table 1) [68,100,101,102]. For instance, miR-155 upregulation and targeting of the KRAS gene lead to inhibition of the Ras/NFkB and reduction of proinflammatory cytokines (IL-17, IL-22, IFN-γ, TNF-α, IL-6 and 2) (Figure 4B, Table 1) [100,101]. Conversely, miR-155 upregulation and targeting of the CD33 gene lead to upregulation of proinflammatory cytokines (IL-1β, IL-8, TNF-α) (Figure 4B, Table 1) [100,101]. miR-155 also promotes production of inflammatory cytokines, such as miR-155’s modulation of toll-like receptors (TLRs) through type I interferons (IFNs), through the NFkB inflammatory pathway (Figure 4C, Table 1) [102,103]. This suggests that miR-155 regulates a network of genes that can either promote or suppress inflammation and cytokine production (Figure 4).

Following microglia activation, miR-155 serves as a pro-inflammatory mediator through the TGFβ pathway by stimulating SMAD4, IL10 and IFNγ (Figure 4C) [95,104,105,106]. Bioinformatics studies revealed that Smad2, a mediator of TGF-β signaling involved in stroke recovery, is a target for miR-155 (Figure 4C) [107]. Interestingly, after stroke, Smad2 and Smad3 are involved in reactive astrogliosis and glial scar formation [145]. Furthermore, miR-155 overexpression decreased SMAD2 protein levels, TGF-β-induced SMAD-2 phosphorylation, and SMAD-2-dependent activation (Figure 4C, Table 1) [107].

Normally, SOCS1 and SHIP1 act as inhibitors to suppress secretion of inflammatory molecules that are activated by the TLR/MyD88, NF-κB, JNK/STAT, PI3K/Akt, and MAPK signaling pathways (Figure 4B, Table 1) [146]. Conversely, miR-155 directly targets and inhibits SOCS1 and SHIP1, promoting cytokine production. In one study, injection of an miR-155 inhibitor (anti-miR-155 miRCURY LNA™, Product# 4101082-001, Exiqon) in stroke-induced mice showed significant decrease in cytokines profiles that were correlated with increased expression of miR-155 targets SOCS1 and SHIP1 (Figure 4B) [146]. miR-155 regulates the Wnt/β-catenin signaling pathway by inhibiting HBP1 (Figure 4C) [72,108]. In IS, a hypoxia-inducible factor (HIF) regulates the transcriptional response to hypoxia [106]. Increased levels of miR-155 decreased HIF-1α mRNA, protein, and transcriptional activity in hypoxic conditions, while inhibition of miR-155 reversed these effects (Figure 4C) [106]. This suggests that miR-155 contributes to a negative feedback loop for HIF-1α activity resolution, resulting in oscillatory behavior of HIF-1α-dependent transcription (Figure 4C, Table 1) [106]. miR-155 enhanced monocyte and macrophage production of proinflammatory cytokines secreting CCL2 (Figure 4C) [104,109]. Additionally, miR-155 indirectly activates the NFkB by inhibiting BCL6, a known inhibitor of NFkB, which has important implications in the signaling of foam cells (Figure 4C) [104,105,109,110,111].

miR-155 and N6-methyladenosine (m6A) RNA methylation in a cerebral ischemia/reperfusion (I/R) injury study using a MCAO model [112] showed that m6A RNA methylation plays a role in mRNA stability, splicing, nuclear retention, and translation efficiency. This process is governed by an m6A methyltransferase complex (METTL3, METTL14, and WTAP, and demethylase FTO (fat mass and obesity-associated protein) and ALKBH5) [112]. The ischemic/reperfusion injury downregulated FTO expression, leading to increased m6A RNA modification of miR-155 which worsened the inflammatory response. This was corroborated by increased infarct volume, neurological deficit score, and IL-1b and TNF-a levels (Figure 4A, Table 1) [112]. Glucocorticoids, commonly used for treating inflammation in the clinic, appear to inhibit miR-155 expression by inhibiting the NFkB (Figure 4C) [130,131]. Notably, the overexpression of miR-155 overwhelmed and reversed the anti-inflammatory effects of glucocorticoids (Figure 4C) (Table 2) [131].

MiR-155’s role in neuroinflammation is closely tied to its interactions with toll-like receptors (TLRs) (Figure 4A, Table 1). miR-155, along with miR-146a/b and miR-21, is thought to regulate TLRs (Figure 4C) [119,147,148,149,150]. When TLRs are triggered by infection, those that recognize pathogen-associated molecular patterns (TLR2, TLR3, TLR4, and TLR9) result in miR-155 upregulation (Figure 4A) [113,119,147,148,149,150]. The TLRs require specific signaling proteins to function, such as MyD88 for TLR2 and TLR9, and TRIF for TLR3 (Figure 4A). MyD88 or TRIF signaling pathways can induce miR-155 expression (Figure 4A) [114].

TNF-alpha also activates miR-155, indicating that IFNs require TNF-alpha signaling in macrophages to increase miR-155 levels (Figure 4A) [113]. Moreover, elevation of TNF-alpha or poly(I:C) can stimulate the JNK pathway leading to further miR-155 upregulation (Figure 4A) [113]. BIC is a gene associated with pathogenesis in leukemias and lymphomas [113]. When investigating regulators of miR-155 levels, BIC mRNA that was produced by upregulation of poly(I:C) or IFN was shown to be involved in miR-155 regulation (Figure 4A) [113]. Additionally, while miR-155 is a downstream target of IFN stimulation, it is an early target gene for poly(I:C) (Figure 4A, Table 1) [113].

In summary, modulation of miR-155 through regulation of target genes can simultaneously influence multiple neuroinflammation signaling pathways. This offers a promising therapeutic strategy for IS, through modulation of uncontrolled inflammation in stroke-injured astrocytes by targeting the suppressor of cytokine signaling (SOCS-1) and M1-microglia (Table 1) [151]. Future stroke treatments should consider use of a miR-155 inhibitor as a potent anti-inflammation agent (Figure 4, Table 1).

## 5. Influence of MiR-155 Modulating Microglia and Astrocytes After Stroke

A critical focus for new therapeutics for stroke today is modulation of neuroprotective supportive cells (e.g., microglia and astrocytes). High levels of miR-155 expression result in activated astrocytes (Table 1). In response to changes in multiple ncRNA levels, monocytes are recruited to the damaged brain tissue and differentiate into M1 pro-inflammatory or M2 anti-inflammatory types (Figure 5) [152]. Increased levels of miR-155 in stroke pre-clinical models ramps up M1 pro-inflammatory polarization, driving up cytokine production and autophagy (Figure 5B, Table 1) [108]. High miR-155 also increased M1 and M2 microglia polarization along with elevated levels of CXCL9, IL-6 and TNF cytokines (Figure 5B, Table 1) [119,120].

Apart from miR-155, other miRNAs (miR-29b, miR-146a, miR-193b, and miR-222), are also elevated during cell differentiation of monocytes to macrophages, suggesting that these miRNAs could also trigger neurotoxic differentiation (Table 3) [120,153]. Several miRNA expression studies looked at the levels of ncRNA in different types of macrophages (M1, M2a, M2b, or M2c) [120]. One showed that the expression of M1 proinflammatory transcripts was increased in THP-1 cells transfected with miR-29b, miR-125a-5p, or miR-155 mimics (Table 3) [120]. Another study using microarray PMA-induced miRNAs (miR-155, mir-222, miR-424 and miR-503) caused cell cycle arrest and partial differentiation, and of these miRNAs two (miR-155 and miR-222) induced G2 arrest and apoptosis (Table 3) [153].

Multiple miRNAs (miR-9, miR-21, miR-24, miR-26a, miR-125a, b, miR-143, miR-145, miR-146a, miR-148, miR-187, miR-223, miR-378-3p, miR-511-3p) modulate macrophage polarization (Table 3) [104,154]. Transfection assays of macrophages with miR-155 mimic resulted in dose-dependent increases in markers CXCL9, which is normally expressed during M1 polarization, IL-6 and TNF, which are expressed during M1 and M2b polarization (Figure 5B, Table 1) [120]. M1 macrophage polarization is induced using IFNγ and TLR4 in vitro by targeting IFNγR, which recruits JAK1/2 to form STAT1/STAT2 heterodimers, which activate NOS2, MHC2 and IL12 [105]. TLR4 activates NFKB and mitogen-associated protein kinase (MAPK) pathways, activating M1-specific transcription factors, with subsequent upregulation of IL12 and downregulation of IL10 (Table 1) [105].

In addition to stimulating M1 polarization, miR-155 inhibits anti-inflammatory M2 polarization by inhibiting IL13RA and C/EBP in the IL13 and IL4 pathways (Figure 5C, Table 1) [107,122,124,125]. Similarly, MafB, a transcription factor important for M2 macrophage polarization, is strongly downregulated by miR-155 (Figure 5C) [124]. Also, IL-13, a pro-M2 cytokine, functions by binding to IL-13 receptor α1 (IL13Rα1), a part of the Type II IL-4 receptor, to activate signal transducer and activator of transcription 6 (STAT6) [122]. miR-155 directly targets and reduces IL13Rα1 protein levels, causing reduced STAT6 activation (Figure 5C, Table 1) [122]. miR-155 also affects IL-13-dependent regulation of SOCS1, DC-SIGN, CCL18, CD23, and SERPINE genes which are involved in differentiating M2 macrophages (Figure 5C, Table 1) [122].

The effects of miR-155 on astrocyte subtype polarization were investigated using DJ-1 (Parkinson disease protein 7, PARK7), an anti-oxidative stress protein with chaperone and signaling functions, and its role in regulating astrocyte neuron survival [126]. Looking for the underlying mechanisms for astrocyte DJ-1 anti-oxidation function and its effects on miR-155 and SHP-1 (Src homology 2 domain-containing phosphatase-1), investigators found that DJ-1 inhibited the transition of astrocytes to the harmful A1 phenotype and promoted A2 polarization, suppressing stroke injury (Table 1) [126]. Furthermore, DJ-1 also interacts with SHP-1 to influence downstream signaling, evidenced by increased SHP-1 elevation with DJ-1 overexpression (Table 1) [126].

In conclusion, miR-155 levels are upregulated by IFNγ and TLR4, both of which drive M1 microglia polarization (Figure 5A, Table 1) [105]. Moreover, miR-155 overexpression reprograms tumor-associated macrophages into M1 macrophages [121], and suppression of SH2-containing inositol-5-phosphatase 1, IL13Rα1 and SMAD2/3, presumed targets for miR-155, promotes M1 macrophage differentiation (Figure 5B, Table 1) [122,123]. Specifically, macrophage M1 to M2 repolarization decreased miR-155 levels while M2 to M1 repolarization increased miR-155, indicating miR-155 plays a vital role in M1 macrophage polarization (Table 1) [121]. Lastly, DJ-1 suppresses miR-155 levels, suggesting that DJ-1 may regulate astrocyte activation via a miR-155/SHP-1 signaling pathway to reduce IS injury (Table 1) [126].

## 6. Proposal to Use NcRNA MiR-155 as a Biomarker for Ischemic Stroke

In the last decade, ncRNAs have been gaining popularity as attractive clinical biomarkers. First, the ease of obtaining accurate miRNA expression level profiles in patient cohorts, combined with expression in pathological tissues, allows for patient risk stratification [169]. Second, quantifying miRNAs expression levels across different tissue types is feasible because biopsies of patient specimens can be processed for qPCR as fresh, frozen or fixed tissue samples which are amenable for analysis with hybridization-based methods or next-generation sequencing [170,171]. Lastly, miRNA liquid biopsies can be conveniently harvested from diseased cells and urine, saliva, cerebrospinal fluid (CSF), synovial fluid, placenta or breast milk [172]. For stroke patients, CSF or blood serum are more likely to be used as the source of miRNAs because of the difficulty associated with resecting brain tissue from living patients. When comparing the technical challenges of quantifying protein versus miRNA biomarkers in CSF or blood samples, miRNA biomarkers are generally much easier to quantify due to the availability of synthetic oligonucleotides that specifically detect PCR products [172]. Furthermore, standardized assays used to analyze miRNAs, such as qRT-PCR, antibody or DNA microarrays, and RNA-sequencing, have high reliability and resolution making them increasingly accessible tools in both academic and clinical settings [173].

There are numerous ncRNAs being investigated as biomarkers in the pipeline. For example, circRNAs are being used as clinical biomarkers for a number of neurological diseases [174,175,176]. In Alzheimer’s disease, circRNAs derived from patients’ CSF, serum, or plasma [177,178], and dysregulated circRNAs were correlated with known disease risk factors (inflammation, dysregulated metabolism, and immune response). Remarkably, for multiple sclerosis, high levels of miR-181c derived from CSF were associated with a conversion from a clinically isolated syndrome to a relapsing–remitting (recovering) multiple sclerosis, while high levels of miR-191-5p and miR-128-3p derived from serum samples were associated with progressive (non-recoverable) multiple sclerosis [179,180]. For stroke, high levels of circRNAs (*circFUNDC1*, *circPDS5B* and *circCDC14A*), were positively correlated with infarct volume (Table 3) [155]. These experiments give a proof-of-principle that profiling miRNA expression levels in CSF or serum could be used clinically to follow disease progression.

The use of miRNAs as clinical biomarkers is also advancing rapidly. For example, miRNAs are being investigated for use in cancer (breast, lung, gastric, pancreatic, biliary tract, neuroendocrine), cardiovascular, neurological, and infectious disease [27,181,182,183]. Investigators have shown that miRNAs also have potential to be used as biomarkers to predict stroke progression [184]. Moreover, miRNA levels in IS brain tissue were profiled (miR-25, miR-125b-2, miR-125b-627, miR-125b-27a, miR-125b-488 and miR-145) as potential biomarkers in cerebral ischemia [157,158]. As stated above, miR-155 was first recognized as a master biomarker when researchers discovered that miR-155 and miR-21 levels were consistently elevated in the serum of diffuse large B-cell lymphoma patients, suggesting that miR-155 levels could predict the progression of the disease [185,186]. Given miR-155’s central role in stroke-related oxidative stress, apoptosis, and neuroinflammation, we and others have proposed that miR-155 is a formidable master biomarker, and future research should address how its modulation can delay the pathological progression of IS [112,117,118,129]. Furthermore, high levels of circulating miR-155 are expected to correlate with ischemic stroke severity in acute settings, and to be indicative of poor long-term outcomes for patients.

### 6.1. Urgent Clinical Need for Time-Sensitive Biomarkers to Manage Stroke

Although miRNAs are accessible in the CSF and blood serum of stroke patients, their clinical application warrants further detailed discussion. As mentioned before, the standard IS treatment depends on a narrow, limited ~4.5-h window for tPA treatment, and a ~6-h window to administer a mechanical thrombectomy. Administration of tPA is counted from the onset of symptoms or last known normal state of the patient. Often, the initial onset of stroke is unknown, putting patients outside the window for therapeutic intervention and excluding patients that may have benefited from standard treatments. To address this challenge, a time-sensitive IS biomarker is urgently needed to reduce or prevent stroke disability.

**Figure 6:**
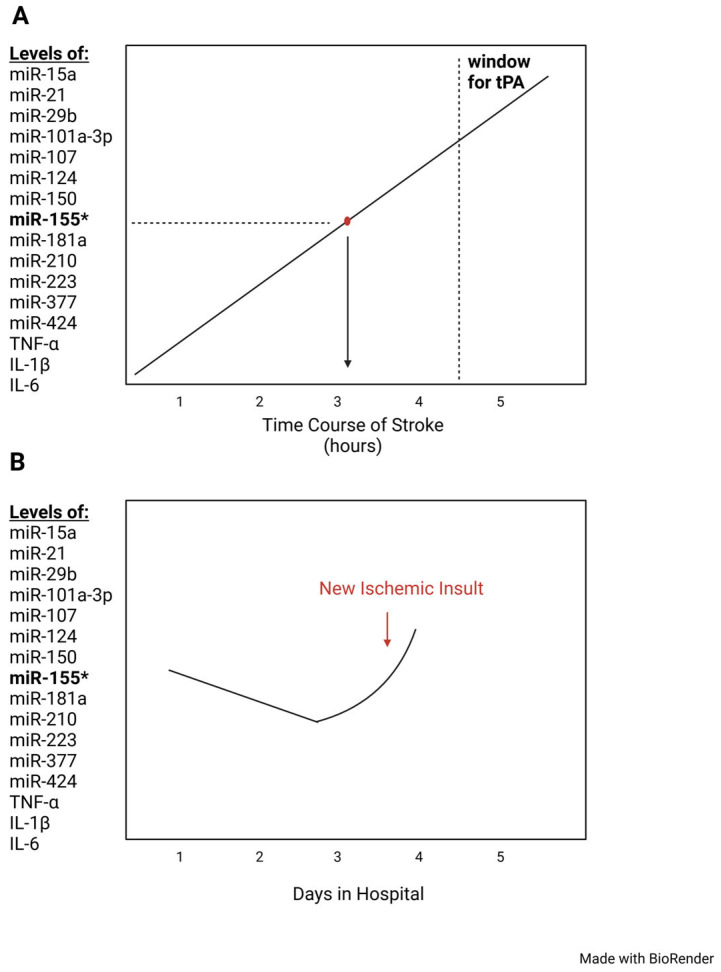
**Use of MiR-155 as early biomarker for stroke.** (**A**) Use of miR-155 as biomarker of stroke timing. If charted out in a graph, clinicians could use the miR-155 level, or levels of time-sensitive markers to determine the length of time the patient has had a stroke to see if they are within the window for tPA therapy. (**B**) Use of miR-155 as biomarker of clinical improvement from stroke. If miR-155 or other markers were to be monitored in the hospital, physicians could use elevations of markers as indicators for new stroke developments that would require intervention. For a more comprehensive list of miRNA biomarkers involved in stroke pathophysiology, please see Tiedt 2018, [168]. The asterisk denotes the master role of miRNA-155 in stroke pathophysiology.

If scientists could identify a reliable time-dependent stroke biomarker whose level correlated to time of stroke onset, physicians could use it in the clinic to rule patients who should or not receive standard of care treatments (Figure 6). As discussed above, miR-155 and accompanying ncRNAs are prime candidates to meet this need. By carefully establishing the timing, dose and administration route for miR-155-based therapeutics, future research could determine a detailed relationship between stroke onset and blood miR-155 levels in different patient stroke populations.

Indeed, such critical time-dependent biomarkers are already used clinically to treat acute heart disease. Creatine kinase (CK-MB), troponin I, and troponin T are time-dependent cardiac biomarkers that have been used for decades to assess the severity of cardiac pathology in myocardial ischemia [187]. CK-MB peaks at 24 h and stabilizes between 48 and 72 h, troponin I peaks at 24 h and stabilizes in 5 to 10 days, and troponin T peaks at 12 to 48 h and normalizes in 5 to 14 days [187]. Investigators have established that miRNAs are promising biomarkers that exhibit time-dependent courses. In a study investigating IS, researchers showed that, after inducing permanent ischemia, levels of miR-101a-3p increased at 30 min and 180 min, and later up to 9 h during transient ischemia [188]. This suggests that miRNA levels such as miR-155, a master regulation of gene expression, could be extremely useful in stroke care (Figure 6).

Finally, it remains to be established if other well-studied, time-sensitive cytokines could be concurrently used clinically to monitor the severity of IS progression. In IS rat models, levels of proinflammatory cytokines TNF-α and IL-1β rise 1 h after a stroke starts [3]. TNF-α reaches its peak at 3 h post-injury, while IL-1β reached its peak at 6 h post-injury. Both cytokines remained elevated up to 2 days after stroke injury [60]. Although TNF-α, a key regulator of neuroinflammation, rises 1–3 h after stroke onset, it may confound stroke severity with other inflammatory conditions. Therefore, a more specific approach is needed.

By carefully measuring the levels of miR-155 and other associated stroke ncRNA at different stroke stages in specific patient populations, we and others propose this could be beneficial for clinical use [189]. We propose that future stroke research profiles must be investigated following time-dependent miR-155 levels in CSF fluid and blood serum in patients between 40 and 50 years old presenting to the emergency room. Ideally, time intervals should measure miR-155 and its precursor gene (10 min, 30 min, 1 h, 2 h, 24 h) (Figure 6).

This kinetic analysis of miR-155 would have great clinical utility because it would allow physicians to determine how far along their patients are in the stroke process and what complications could ensue allowing more personalized decision making to manage stroke in clinical settings. Moreover, a reliable group of time-sensitive biomarkers would thereby help physicians rule in patients to receive treatment with tPA or a mechanical thrombectomy. Another use of miR-155 biomarkers would be to monitor improvement in stroke patients after standard of care treatment, or after taking a miR-155 inhibitor, discussed below (Section 7.2). This information would alert physicians of new strokes or ineffective treatment, allowing them to intervene early to reduce the disability and mortality of stroke patients.

Future research should systematically determine the profile levels of miR-155 in different stroke presentations, considering a patient’s clinical history and adverse effects. Clinical trials should address how stroke outcomes improve after timely administration of miR-155 antagomirs, engineered oligonucleotides designed to complementarily bind and silence miRNAs, in cohort studies. Together, these studies will help determine miR-155’s potential to be used as a biomarker in stroke clinical diagnosis, prognosis and treatment.

### 6.2. Artificial Intelligence and Stroke Biomarkers

In treating IS injury, the timeliness of diagnosis and intervention are crucial to preserve brain tissue function and to maximize long-term cognitive, behavioral, and motor outcomes for patients. Therefore, multiple efforts, including integrating the clinical use of artificial intelligence (AI), are underway to improve timely diagnosis of stroke and outcomes. Apart from miR-155, other adjuvant stroke-specific biomarkers could enhance the predictive value of AI. In a proteomics stroke study, researchers identified ICAM-2, STXBP5, PLGLA, C3, and IGHV3-64 as candidate biomarkers in blood, yielding a high (75% to 88%) sensitivity for identifying stroke patients [190]. Remarkably, it was found that glial fibrillary acidic protein (GFAP) was sensitive in differentiating IS from hemorrhagic stroke at 3 h and 24 h after stroke onset [191]. Antithrombin III (ATIII), fibrinogen, and ischemia-modified albumin (IMA) are being validated pre-clinically for this purpose [192].

AI is also being analyzed for its potential to improve the routine stroke treatment paradigm, by image differentiation of ischemic and hemorrhagic stroke, large vessel detection, and early CT score grading [193]. Studies are focusing on the use of AI in predicting outcomes for patients based on a combination of clinical parameters [194]. Moreover, progress in predicting reliable stroke diagnosis and treatment requires standardized and validated protocols to holistically analyze clinical history and social determinants of health [194]. Lastly, machine learning algorithms identify large vessel occlusion (LVO) on CT scans to screen patients who are eligible for endovascular interventions with thrombectomy [195].

## 7. Discussion

### 7.1. Central Role of MiR-155: Could the Pathology of Acute Ischemic Stroke Be Reversed by miRNA-155 Specific Inhibitors

miR-155 plays an intricate role in the pathophysiology of acute IS through its modulatory effects encompassing oxidative stress, apoptosis, neuroinflammation, and polarization of microglia and astrocytes (Figure 1B). In regulating oxidative stress, miR-155 affects the expression of many key pathways. For instance, in the lncRNA OIP5-AS1/miR-155-5p/IRF2BP2 axis, lncRNA OIP5-AS1 interacts and represses miR-155-5p, preventing miR-155-5p from binding its target IRF2BP2 who suppresses oxidative stress (Figure 2A) [129]. During acute stroke, higher levels of miR-155-5p overwhelm the inhibitory effects of lncRNA OIP5-AS1, allowing miR-155-5p to bind its target IRF2BP2 to enhance oxidative stress [129]. Given miR-155’s impact on oxidative stress, the use of miR-155 inhibitors and their outcomes on ischemic injury have been widely investigated. Use of miR-155-5p inhibitors in NSCs significantly reduced oxidative stress and inflammation, decreased infarct volume, and improved neurobehavioral outcomes in IS models, suggesting miR-155 inhibitors could be neuroprotective against cerebral infarction if applied to clinical settings (Figure 2B) [132]. Importantly, these findings were confirmed when miR-155 inhibitors reduced upregulation of pro-inflammatory cytokines (PICs) into the hippocampus, decreased oxidative stress, and improved neurological severity score and brain edema after ischemia, suggesting miR-155 inhibitors may have a potential therapeutic role in treating oxidative stress triggered by ischemia (Figure 2C) [133]. In another mouse MCAO model of IS, use of intravenous injections of miR-155 inhibitor resulted in reduced infarct size, decreased neuron damage, and improved functional recovery in the inhibitor-injected mice, suggesting that future miR-155 inhibitors could be therapeutically effective by intravenous routes [196].

Data from hypoxic–ischemic brain preclinical studies show that miR-155 enhances apoptosis and inflammation via inhibiting SIRT1 in neonatal rats with HIBD (Figure 3B) [115]. Also, miR-155-5p directly targets DUSP14 by regulating the NF-κB and MAPKs signaling pathways to worsen apoptosis (Figure 3A) [116]. Additionally, the increase in miR-155 levels triggered by IS activates the TLR4/MyD88 signaling pathway, worsening apoptosis of vascular endothelial cells (Figure 3B) [117]. While miR-155 promotes apoptosis, its effects can be overcome by modulation of downstream signaling pathways. For example, in the Oprm1/miR-155/GATA3 axis-regulated apoptosis, overexpression of lncRNA Oprm 1 alleviated apoptosis of IS, significantly decreasing the infarct size and improving neurological score (Figure 3C)[134]. Taken together, these findings suggest that miR-155 is a prime regulator of apoptosis and could potentially serve as a target for stroke-specific development of RNA-therapeutic inhibitors.

We describe here miR-155’s potent modulatory role in neuroinflammation through regulation of NFkB, as it promotes production of inflammatory cytokines that worsen stroke injury (Figure 4C, Table 1). The role of miR-155 and neuroinflammation is closely tied to its interactions with toll-like receptors (TLRs) (Figure 4A, Table 1). miR-155 likely collaborates with miR-146a/b and miR-21 to regulate TLR pathways to oversee inflammation (Figure 4C, Table 1) [119,147,148,149,150]. It is therefore possible that using a miR-155 inhibitor may serve as an adjuvant to reduce inflammation triggered by IS. miR-155 also interferes with the neuroprotective effects of microglia and astrocytes in the brain during ischemic injury (Figure 5, Table 1). miR-155 promotes M1 pro-inflammatory microglia polarization and inhibits M2 anti-inflammatory polarization to reverse microglia normal neuroprotection (Figure 5B,C, Table 1) [108,120,124]. Similarly, miR-155 promotes A1 pro-inflammatory astrocyte activation which is reversed by DJ-1 suppression of miR-155 through the miR-155/SHP-1 signaling pathway (Table 1). Lastly, DJ-1 suppresses miR-155 levels, suggesting that DJ-1 may regulate astrocyte activation via a miR-155/SHP-1 signaling pathway to reduce IS injury [126].

### 7.2. MiR-155 Inhibitors (anti-RNA) as Therapeutic for Ischemic Stroke: Antisense Oligonucleotides

As stated above, the approach of using miRNA inhibitors as therapeutics is being considered for many diseases [197,198,199]. Given miR-155’s role in regulating acute stroke pathogenesis in oxidative stress, apoptosis, neuroinflammation, and microglia/astrocyte polarization, a miR-155 inhibitor antisense oligonucleotide (ASO) could be used in combination with standard stroke therapy with tPA or thrombectomy. Cobomarsen (also called MRG-106) is an oligonucleotide miR-155 inhibitor used for treating cutaneous T-cell lymphoma [200]. Currently, a phase I clinical trial using Cobomarsen has shown that the miR-155 inhibitor was well-tolerated in patients, indicating it could be used potentially in other disease processes, such as IS [30].

MicroRNA (miRNA) inhibitors, or anti-microRNAs (anti-RNAs), are the antisense oligonucleotide (ASO) which can bind to its complementary miRNA to inhibit its function [201]. In pre-clinical research studies, there are already several miR-155 inhibitors in use [202,203,204]. miRNAs are not currently available for use clinically, but there are several clinical trials assessing their safety and effectiveness. MRX34, a mimic of the tumor suppressor miRNA miR-34a for cancer, and an anti-miR targeting miR-122, is being evaluated to treat hepatitis C [205,206]. Interestingly, miR-155 inhibitors are also being investigated in clinical trials for rheumatoid arthritis (RA), an autoimmune disease with elevated miR-155 and M1 macrophages [207]. In a clinical trial for RA, transfection of healthy monocytes with miR-155 promoted M1 microglia, while transfecting RA monocytes with antagomir miR-155 promoted anti-inflammatory M2 microglia [207].

While miR-155 and other miRNA inhibitors could have therapeutic benefit in treating ischemic stroke, it is important to discuss possible complications in using these inhibitors clinically. MiRNAs, like miR-155, are master regulators in multiple disease pathways, so miR-155 inhibitors could have potential off-target effects that need to be thoroughly investigated before they can be used in clinical settings. Additionally, miRNAs are thought to be interconnected in their regulations of pathways, therefore miR-155 inhibitors could trigger compensatory mechanisms by other miRNAs, such as miR-21 and miR-124, that could influence therapeutic outcomes. Future research should address the nuances of miR-based therapeutics for all stroke types.

### 7.3. MiR-155 as Biomarker for Ischemic Stroke

As mentioned above, miRNAs today are being assessed in clinical practice to monitor cancer, atrial fibrillation, endocrine disorders, heart failure, lung diseases, and neurological disorders (traumatic brain injury, Alzheimer’s Disease, and Parkinson’s disease) [187,208,209,210,211,212,213,214,215,216,217]. However, no validated clinical biomarker currently exists, to our knowledge, for IS. In the clinic, stroke diagnosis is reached by provider clinical evaluation of CT/MRI imaging [218].

A biomarker for acute IS must reliably measure levels across different stages of stroke (Figure 6). It is possible to evaluate TNF-α, an inflammatory cytokine, during IS injury as it is known to initially peak in the first 1–3 h, and again after 24–36 h [60,61]. In stroke patients, the rise in TNF-α can be measured 6–12 h from symptom onset [219]. Additionally, a decrease in TNF-α concentration within 72 to 144 h after stroke was correlated with clinical improvement [220]. IL-1β has been detected within 1 h after ischemic brain injury, and IL-6 was increased within a few hours after onset of ischemia and up to 90 days after stroke [221,222]. miRNAs can also be time-sensitive markers. miR-101a-3p, another miRNA being considered as a biomarker for IS, is time-sensitive. During IS, miR-101a-3p levels increased at 30 min and 180 min, and after inducing permanent ischemia remained elevated up to 9 h after ischemia (Figure 6) [188].

Given miR-155’s close regulatory role in the pathophysiology of a stroke, it is imperative that the levels of miR-155 be systematically profiled. Research would determine the feasibility at stroke onset, and as a time-sensitive biomarker capable of indicating clinical progression. A combination of miR-155 with other miRNAs would likely be useful as biomarkers to monitor acute IS progress. To tailor stroke treatment to a particular patient population, future research should determine the combination of miRNAs involved in IS that could potentially serve as biomarkers (Table 3). Candidates include miR-181c, miR-124, miR-126, miR-130, miR-181, miR-107, miR-15a, miR-16–1, miR-133, miR-1906, miR-99a, miR-497, miR-424, and miR-210 [3,156,157,158,159,160,161,162,163,164,165,166,167]. We refer the reader to other reviews that have discussed mechanistic details on how miR-155-associated miRNAs might also be included to investigate biomarker panels for specific patient populations [168,223,224,225].

### 7.4. Outstanding Challenges and Future Stroke Research Directions

While the proposal of miR-155 as a time-sensitive biomarker for ischemic stroke is promising, there remains a need to critically discuss clinical challenges. Concerns for patient safety remain in determining miR-155 as a time-sensitive biomarker for ischemic stroke in its expression across patient populations with different demographics and co-morbidities, with their expression in different number and stroke type presentation. Next, sample stability of miR-155 in CSF versus serum samples needs to be carefully addressed.

As such, future studies should investigate large-scale patient cohorts analyzing the levels of miR-155 and other miRNAs to strengthen miR-155’s validity as a time-sensitive biomarker for ischemic stroke. In the clinic, standard stroke protocols exist where once a “*stroke code*” has been activated, blood samples are often analyzed after performing head CT imaging and administering tPA [65]. Therefore, if a biomarker panel of miR-155 and other miRNAs were validated for clinical use, this could conveniently be added to the standard blood work for stroke patients to transform stroke outcomes. The miR-155 biomarker values could be monitored to evaluate stroke progression, similar to protocols in place for myocardial ischemia and troponins.

While Cobomarsen (miR-155 inhibitor) has the potential to be incorporated into clinical standard stroke regimens as therapeutics, there are several barriers that need to be considered. First, the ability of miR-155 inhibitors to penetrate the blood–brain barrier using EVs or carrier liposomes must be determined. Next, the level of uptake of miR-155 into neurons and glial cells, and the lifetime of their therapeutic outcomes on reducing oxidative stress, apoptosis, infarct volume and neuroinflammation, must be established. Finally, the long-term cognitive and motor outcomes must be carefully followed. Similarly, potential off-target effects, interactions with other miRNAs, and drug interactions need to be investigated before miR-155 inhibitors can be used in a clinical routine setting in conjunction with standard stroke therapies.

## 8. Conclusions

Stroke continues to be a significant cause of mortality and disability worldwide and is difficult to treat clinically to avoid long-term disability due to the time-restrictive nature of current standard treatments. Current clinical treatment for IS is focused on re-establishing blood flow and oxygenation to the brain using tPA and thrombectomy. However, these treatments do not address the underlying pathophysiology of stroke injury, including oxidative stress, apoptosis and neuroinflammation. Reliable stroke therapeutics that target the molecular and cellular deficits caused by stroke are needed to reverse neurotoxicity and disability.

miR-155 plays a key role in promoting the pathogenesis of IS, including oxidative stress, apoptosis, neuroinflammation, and microglia/astrocyte polarization, making miR-155 inhibitors attractive therapeutics to manage the acute phase of stroke that could be combined with current standard IS therapies (e.g., tPA and thrombectomy). Given the complex and overlapping regulation of stroke pathophysiology by ncRNAs, future research should focus on establishing the critical time frame to administer miR-155 to patients, with or without adjuvant miRs. This regimen will determine whether ncRNA-based stroke therapeutics are validated in the clinic as effective agents for monitoring stroke onset and progression (Table 4) [168].

## Figures and Tables

**Table 1:** MiR-155 Roles in Stroke Pathology.

Signaling Pathway	miR-155 Levels	Key Target Gene	Function	Reference
**Neuroinflammation**				
Ras/NFkB	Upregulated	KRAS	Upregulation of miR-155 target the KRAS gene inhibiting Ras/NFkB signaling and reducing proinflammatory cytokines (IL-17, IL-22, IFN-γ, TNF-α, IL-6 and 2).	[100,101]
NFkB	Upregulated	CD33	miR-155 upregulation targets the CD33 gene leading to increased levels of proinflammatory cytokines (IL-1β, IL-8, TNF-α).	[100,101]
NFkB	Upregulated	N/A	miR-155 upregulation leads to increased inflammation through NFkB pathway activation, causing high levels of TLR-signaling inflammatory cytokines and type I interferons (IFNs).	[102,103]
NFkB and TGFβ	N/A	MIRHA155 Gene	Regulatory elements in the MIRHA155 gene are stimulated by IL10 and IFNγ; and modulated by the NFkB pathway and the TGFβ pathway through SMAD4.	[95,104,105,106]
TGFβ	Upregulated	SMAD2	miR-155 targets SMAD2, a TGFβ signaling mediator, to decrease SMAD2 protein levels, TGF-β-induced SMAD-2 phosphorylation, and SMAD-2-dependent activation.	[107]
TLR/MyD88, NF-κB, JNK/STAT, PI3K/Akt, and MAPK	Upregulated	SOCS1 and SHIP1	miR-155 targeting of SOCS1 and SHIP1 activates the TLR/MyD88, NF-κB, JNK/STAT, PI3K/Akt, and MAPK signaling pathways to promote secretion of inflammatory cytokines and chemokines.	[72,108]
Wnt/β-catenin	Upregulated	HBP1	miR-155 regulates the Wnt/β-catenin signaling pathway by inhibiting HBP1.	[72,108]
Ischemic stroke	Upregulated	hypoxia-inducible factor (HIF)	Increased miR-155 levels decrease HIF-1α mRNA, protein, and transcriptional activity in hypoxic conditions.	[106]
Monocyte/macrophage	Upregulated	N/A	miR-155 enhanced monocyte and macrophage production of proinflammatory cytokine CCL2.	[104,109]
NFkB	Upregulated	N/A	miR-155 inhibits BCL6, an inhibitor of NFkB.	[104,105,109,110,111]
FTO/m6A RNA methylation, IL-1b/TNF-a	Upregulated	N/A	Downregulation of FTO expression increases N6—methyladenosine (m6 A) RNA modification of miR-155, which worsens infarct volume, neurological deficit, and inflammatory IL-1b and TNF-a levels.	[112]
TLR	Upregulated	N/A	TLR2, TLR3, TLR4, and TLR9 induce miR-155 elevation.	[113]
TLR	Upregulated	N/A	MyD88 and TRIF signaling pathways induce miR-155 expression. MyD88 is necessary for TLR2 and TLR9 signaling, and TRIF for TLR3 signaling.	[114]
TNF-alpha	Upregulated	N/A	TNF-alpha is an miR-155 inducer. IFNs require TNF-alpha in macrophages to increase miR-155 levels.	[113]
JNK Pathway	Upregulated	N/A	TNF-alpha or poly(I:C) stimulation of the JNK pathway increases miR-155 upregulation.	[113]
BIC	Upregulated	BIC gene	Upregulation of poly(I:C) or IFN- increases BIC mRNA involved in miR-155 upregulation. miR-155 is a downstream target of IFN- and an early gene target for poly(I:C).	[113]
**Apoptosis**				
Apoptosis	Upregulated	SIRT1	miR-155 targets and inhibits SIRT1, promoting apoptosis.	[115]
Apoptosis	Upregulated		miR-155-5p directly targets and inhibits DUSP14 by binding the 3′UTR, thereby preventing DUSP14 from inhibiting the NF-κB and MAPKs signaling pathways, resulting in increased apoptosis.	[116]
Apoptosis (TLR4/MyD88)	Upregulated		Increased miR-155 levels activate the TLR4/MyD88 signaling pathway, worsening the inflammatory response following stroke.	[117]
Apoptosis (Rheb/mTOR)	Upregulated		miR-155-5p promotes apoptosis by suppressing Rheb expression and by promoting NLRP3-mediated inflammasomes.	[118]
**Microglia**				
M1 microglia polarization	Upregulated		High miR-155 levels increase cytokine production, autophagy, and M1 pro-inflammatory polarization.	[108]
M1 and M2 microglia polarization	Upregulated		High levels of passenger strand miR-155 were detected in M1 and M2 microglia polarization.	[119]
M1 and M2b polarization	Upregulated		Transfection of macrophages with miR-155 increased CXCL9 (M1 marker), and IL-6 and TNF (M1 and M2b markers).	[120]
M1 macrophage polarization	Upregulated		IFNγ and TLR4 induce M1 macrophage polarization in vitro, and miR-155 level are upregulated by IFNγ and TLR4.	[105]
M1 macrophage polarization	Upregulated		miR-155 overexpression reprogrammed tumor-associated macrophages into M1 macrophages.	[121]
M1 macrophage polarization	Upregulated		miR-155 targets and suppresses SH2-containing inositol-5-phosphatase 1, IL13Rα1 and SMAD2/3, promoting M1 macrophages.	[122,123]
M2 macrophage polarization	Upregulated		miR-155 inhibits the anti-inflammatory M2 polarization by inhibiting IL13RA and C/EBP in the IL13 and IL4 pathways.	[107,122,124,125]
M2 macrophage polarization	Upregulated		miR-155 downregulated MafB, a transcription factor important for M2 macrophage polarization.	[124]
M2 macrophage polarization	Upregulated		IL-13, a pro-M2 cytokine, functions by binding to IL-13 receptor α1 (IL13Rα1), a part of the Type II IL-4 receptor, to activate signal transducer and activator of transcription 6 (STAT6). miR-155 directly targets and reduces IL13Rα1 protein levels, causing reduced STAT6 activation.	[122]
M2 macrophage polarization	Upregulated		miR-155 impacts IL-13-dependent regulation of SOCS1, DC-SIGN, CCL18, CD23, and SERPINE genes involved in differentiating M2 macrophages.	[122]
Astrocyte polarization	Upregulated		DJ-1 suppresses miR-155, regulating astrocyte activation via miR-155/SHP-1 signaling pathway. DJ-1 inhibits transition of astrocytes to harmful A1 subtypes, promoting beneficial A2 polarization.	[126]

**Table 2:** MiR-155 Inhibitors in Management of Ischemic Stroke.

	miR-155 Levels	Principal Target Gene	Function	Reference
NFkB	Downregulated	N/A	miR-155 overexpression reversed the anti-inflammatory effects of glucocorticoids, while miR-155 inhibition restored them.	[130,131]
Oxidative Stress	Downregulated	N/A	lncRNA OIP5-AS1 interacts and represses miR-155-5p, preventing miR-155-5p from binding its target IRF2BP2, which then suppresses oxidative stress.	[129]
Oxidative Stress	Downregulated	N/A	miR-155-5p directly targets HIF-1α and negatively regulates its expression, promoting oxidative stress. miR-155-5p inhibitor in NSCs reversed miR-155-5p’s inhibition of HIF-1α, resulting in reduced oxidative stress and inflammation, decreased infarct volume and improved neurobehavioral outcomes.	[132]
Oxidative Stress	Downregulated	N/A	miR-155 inhibitor significantly reduced upregulation of hippocampus pro-inflammatory cytokines (PICs), decreased oxidative stress, and improved neurological severity score and reduced brain edema.	[133]
Apoptosis	Downregulated		Overexpression of lncRNA Oprm1 decreases apoptosis, through a lncRNA Oprm1/miR-155/GATA3 axis, by significantly decreasing infarct size and improving neurological score.	[134]
Apoptosis	Downregulated		High cerebral infarct volumes and apoptosis were associated with increased miR-155 and decreased Rheb, mTOR, and pS6K, while treatment with miR-155 inhibitors were protected with reduced apoptosis and increased Rheb, mTOR, and p-S6K expression	[135]

**Table 3:** MiR-155 vs Other NcRNAs involved in Cellular Processes of Ischemic Stroke.

NcRNA	Function in Ischemic Stroke	Reference
miR-155 miR-146a/b miR-21	miR-155, miR-146a/b and miR-21 create a trinity that regulate TLR pathways to control inflammation in stroke	[119,147,148,149,150,151]
miR-155 miR-29b miR-146a miR-193b miR-222	miR-155, in addition to miR-29b, miR-146a, miR-193b, and miR-222, are elevated in monocyte to macrophage differentiation.	[120,153]
miR-9 miR-21 miR-24 miR-26a miR-125a, b miR-143 miR-145 miR-146a miR-148 miR-187 miR-223 miR-378-3p miR-511-3p	miR-9, miR-21, miR-24, miR-26a, miR-125a, b, miR-143, miR-145, miR-146a, miR-148, miR-187, miR-223, miR-378-3p, miR-511-3p have been reported to play a role in macrophage polarization.	[104,154]
circFUNDC1 circPDS5B circCDC14A	High levels of circFUNDC1, circPDS5B and circCDC14A were found to be positively correlated with infarct volume in acute ischemic stroke.	[155]
miR-15a/16-1 cluster	Endothelium-targeted deletion of the miR-15a/16-1 cluster ameliorates blood–brain barrier dysfunction in ischemic stroke and poststroke angiogenesis	[156]
miR-181c	miR-181 suppresses TNF-α expression in post-ischemic neuronal damage.	[157]
miR-155	miR-155 exerts both pro- and anti-inflammatory effects by targeting mediators of inflammatory signaling— SHIP1, SOCS1, SMAD2 and TAB2.	[158]
miR-126	Increased in endothelial cell or CV functions in ischemic stroke.	[159]
miR-130	Increased in angiogenesis in ischemic stroke.	[159]
miR-181	Increased in infarct core and decreased in penumbra after focal ischemia. miR-181 was shown to sensitize cells to apoptosis by reducing Bcl-2.	[160]
miR-107	Increased miR-107 levels may regulate post-ischemic stroke angiogenesis.	[161]
miR-15a/16–1	miR-15a/16–1 repress pro-angiogenic factors VEGFA and FGF2 and their receptors VEGFR2 and FGFR1..	[156]
miR-133	Overexpressing MSCs further stimulates and increases exosomes’ release from astrocytes, possibly by downregulating the RABEPK expression..	[162]
miR-1906	Is increased in glial cells and decreased in neurons. miR-1906 is involved in abolishment of TLR4 protein expression and could ameliorate brain injury in stroke.	[163]
miR-99a	miR-99a prevented apoptosis and blocked cell cycle progression in neuro-2a cells	[164]
miR-497	miR-497 promotes ischemic neuronal death by negatively regulating anti-apoptotic proteins bcl-2 and bcl-w.	[165]
miR-424	miR-424 prevents ischemic brain injury by suppressing microglia activation.	[166]
miRNA-210 + HIF-1α	HIF-1α induces miR-210 which could prevent apoptosis and induce angiogenesis.	[167]
miR-124, miR-223, miR-107, miR-181a	Involved in regulating excitotoxicity in stroke.	[168]
miR-21, miR-210, miR-424, miR-29b, miR-124, miR-15a, miR-181a	Involved in regulating apoptosis and programmed cell death in stroke.	[168]
miR-29b, miR-124, miR-150, miR-155	Involved in promoting blood–brain barrier breakdown after stroke.	[168]
miR-21, miR-124, miR-223, miR-424, miR-15a, miR-155, miR-181a, miR-210, miR-377	Involved in promoting inflammation in stroke.	[168]
miR-124	Involved in protective effects against stroke including angiogenesis and neurogenesis, and damaging effects of stroke including excitotoxicity, programmed cell death, blood–brain barrier breakdown, and inflammation.	[168]

**Table 4:** Future Directions for MiR-155 in Diagnosis of Ischemic Stroke.

miR-155 Research Future Directions	Recommendations
Profiling miR-155 stability in patients	Test the stability and abundance of miR-155 in CSF and blood serum IS patient samples as compared to healthy controls at different stages of the disease.
Measuring expression miR-155 levels variability in different patient populations with severity of stroke types	Establishing miR-155 differential expression levels in different patient populations.
Need to evaluate efficacy in large scale cohort studies	Analyze beneficial/adverse effects of miR-155 administration in large IS patient cohorts.
Refine integration of miR-155 with IS standard of care protocols	Analysis of existing stroke diagnostic protocols and potential incorporation of miR-155 into diagnosis algorithm

## Data Availability

Not applicable.

## Abbreviations

alteplase (tPA), anti-microRNAs (anti-RNAs), antisense oligonucleotide (ASO), antithrombin III (ATIII), artificial intelligence (AI), central nervous system (CNS), cerebrospinal fluid (CSF), choroid plexus epithelial (CPE), circular RNA (circRNA), creatine kinase (CK-MB), extracellular vesicles (EVs), fat mass and obesity-associated protein (FTO), glial fibrillary acidic protein (GFAP), 8-hydroxy-2′-deoxyguanosine (8-OHdG), hypoxia-inducible factor 1α (HIF-1α), hypoxic–ischemic brain damage (HIBD), IL-13 receptor α1 (IL13Rα1), interferon regulatory factor 2 binding protein 2 (IRF2BP2), ischemia-modified albumin (IMA), ischemia/reperfusion (I/R), ischemic stroke (IS), isoprostaglandin F2α (8-iso PGF2α), large vessel occlusion (LVO), long non-coding RNA (lncRNA), malondialdehyde (MDA), microRNA (miRNA), middle cerebral artery occlusion (MCAO), middle cerebral artery occlusion/reperfusion (MCAO/R), mitochondria permeability transition pore (MPTP), mitogen associated protein kinase (MAPK), neural stem cell (NSC), N6—methyl adenosine (m6A), N-methyl-D-aspartate (NMDA), nicotinamide adenosine nucleotide (NAD), non-coding RNA (ncRNA), nuclear factor-κB (NF-κB), oxygen-glucose deprivation/reoxygenation (OGD/R), Parkinson disease protein 7, PARK7 (DJ-1), pro-inflammatory cytokine (PIC), reactive oxygen species (ROS), reduced superoxide dismutase (SOD), rheumatoid arthritis (RA), signal transducer and activator of transcription 6 (STAT6), silent information regulator, sirtuin 1 (SIRT1), Src homology 2 domain-containing phosphatase-1(SHP-1), toll-like receptors (TLRs), vascular endothelial cell (VEC).
